# Transpancreatic biliary sphincterotomy for biliary access is safe also on a long-term scale

**DOI:** 10.1007/s00464-019-07364-y

**Published:** 2020-01-28

**Authors:** Vilja Koskensalo, Marianne Udd, Mia Rainio, Jorma Halttunen, Matias Sipilä, Outi Lindström, Leena Kylänpää

**Affiliations:** 1 Abdominal Center, Helsinki University Hospital, Haartmaninkatu 4, 00029 HUS Helsinki, Finland; 2 University of Helsinki, Helsinki, Finland

**Keywords:** Transpancreatic biliary sphincterotomy, Endoscopic retrograde cholangiography, Post-endoscopic retrograde cholangiopancreatography pancreatitis

## Abstract

**Background:**

Transpancreatic biliary sphincterotomy (TPBS) is an advanced cannulation method for accessing common bile duct (CBD) in endoscopic retrograde cholangiopancreatography (ERCP). If CBD cannulation is difficult, an endoscopist can open the septum between the pancreatic and biliary duct with a sphincterotome to gain access. Long-term results of this procedure are unclear. We wanted to evaluate the short- and long-term complications of TPBS on patients with native papilla and benign indication for ERCP.

**Patients and Methods:**

ERCPs performed in Helsinki University Hospital between 2007 and 2013 were reviewed. The study group comprised 143 consecutive patients with TPBS and 140 controls (CG). Data were collected from patient records and a phone survey was performed as a follow-up ≥ 4 years after the index ERCP.

**Results:**

Post-ERCP pancreatitis (PEP) developed in seven patients (4.9%) in TPBS and one patient (0.7%) in CG (*p* = 0.067). The rates of other acute complications were similar between the groups. ERCP ended with no access to CBD in four cases (2.8%) in TPBS. The median length of follow-up was 6 years in TPBS and 7 years in CG. During this period, three patients (2.1%) in TPBS and six patients (4.3%) in CG suffered from acute pancreatitis (AP) (*p* = 0.238). One (0.7%) patient in CG and none in TPBS developed chronic pancreatitis (CP). Abdominal pain was suffered by ten patients (6.9%) in TPBS and twelve patients (8.6%) in CG daily, whereas by six patients (4.2%) in TPBS and twelve patients (8.6%) in CG weekly.

**Conclusion:**

TPBS is a useful procedure, with acceptable complication rates. No significant difference occurred between the groups when evaluating the short-term or long-term complications with a follow-up period of four to 10 years. Additionally, no significant differences occurred in upper abdominal pain, episodes of AP, or development of CP.

ERCP (endoscopic retrograde cholangiopancreatography) is one of many methods to examine and treat diseases of the bile duct and the pancreatic duct. Cannulation of the papilla (papilla Vater) is often performed with a guidewire and a sphincterotome. According to the European Society of Gastrointestinal Endoscopy (ESGE) guidelines, biliary cannulation is defined as difficult if cannulation lasts longer than five minutes, success requires more than five attempts, or the guidewire accidently passes the pancreatic duct at least twice [1]. Therefore, additional cannulation methods are often needed in a difficult cannulation [1, 2]. Regardless of all methods, final failure occurred in 2.6–18.0% of all ERCP procedures [2–4].

One advanced additional cannulation method is transpancreatic biliary sphincterotomy (TPBS). TPBS was first described by Goff in 1995 [5]. This method involves opening the septum between the common bile duct (CBD) and pancreatic duct (PD) if the guidewire unintentionally enters the PD. The incision is performed with the sphincterotome directed towards the axis of the CBD, in the 11 or 12-o’clock direction, to detect the bile duct orifice to cannulate CBD [1, 5, 6]. The rate of successful CBD cannulation after TPBS is 85–100% [1]. If TPBS alone is not successful, an additional needle knife (NK) cut can be performed obliquely towards 10 o`clock from the superior end of the previous TPBS [7].

ERCP is an invasive procedure with an overall complication rate of approximately 4–11% [8–11]. The most common complication is post-ERCP pancreatitis (PEP). Among unselected ERCP patients, the PEP rate was 1.8–4.2% [8–10]. In a native papilla cannulation, the PEP rate was 5.3% [2] and is rather high 10.8–14.9% in difficult cannulation [1–3, 11]. When using TPBS as a rescue method in cases of difficult cannulation, the PEP rate was 7.1–10.4% [2, 7, 12–14]. Other possible ERCP complications include cholangitis or other infection (1.4%), bleeding (1.3%) and perforation of bowel or biliary tract (0.1–0.6%) [9, 15].

PEP was mild or moderate in 90% of the cases. However, 10% of the PEP patients suffered from a severe form of the disease and risk of death among these patients is 3–10% [12, 16]. The risk of death in all ERCP procedures is 0.06–0.2% [9, 11, 17].

Risk factors for PEP can be classified as patient- or procedure-related factors. Patient- related factors include a patient’s young age, female sex, history of PEP, recurrent acute pancreatitis (AP), diseases such as primary sclerosing cholangitis (PSC) and sphincter of Oddi dysfunction (SOD) [12, 18, 19]. Procedure-related risk factors include difficult cannulation, accidental cannulation of PD, long-lasting ERCP procedures, biliary balloon sphincteroplasty, minor papilla sphincterotomy, pancreatic duct injection, certain cannulation methods (e.g., TPBS or precut sphincterotomy) and trainee involvement [12, 18, 19]. These risk factors increase the risk of PEP synergistically [12]. In addition, ERCP procedures can be graded according to criteria concerning degrees of difficulty [20, 21], which is helpful when comparing the risk of complications.

This study aimed to evaluate acute and long-term complications of TPBS. The primary outcomes were development of episodes of PEP, AP during the follow-up period, development of chronic pancreatitis (CP) and post-sphincterotomy stenosis. The secondary outcomes were other ERCP complications and re-ERCPs performed during the follow-up period.

## Patients and methods

The study design was a retrospective case–control study with a phone survey as a cut point. Calculation of the sample size involved estimated PEP rates of 2.2% and 10.4% in regular wire-guided cannulation and in TPBS, respectively (power = 0.8, α = 0.05). According to this calculation, we aimed to both include a minimum of 137 participants in both groups and have a long-term follow-up period lasting at least four years after the first ERCP.

### Patients

We reviewed the ERCPs performed between January 2007 and December 2013 in the Endoscopy Unit of the Helsinki University Hospital (HUS). Indications of the needs for ERCP were defined from the procedure data. The exclusion criteria identified were primary sclerosing cholangitis (PSC), chronic pancreatitis (CP), biliary or pancreatic malignancies, being aged under 18 and deceased patients. Remaining in the study after exclusion was 170 consecutive TPBS patients with native papilla. Indications for ERCPs in this study were CBD stones, benign biliary stricture, problems related to post-liver transplantation (stricture), bile leakage after cholecystectomy and SOD. For these TPBS patients, we searched for 170 consecutive age- (+ /− 10 years) and sex-matched controls with the same inclusion criteria but having successful biliary access without requiring TPBS. Of these groups, 143/170 (84.1%) TPBS patients and 140/170 (82.6%) patients in control group (CG) participated in the phone survey and comprised the final study groups.

Patients and controls received a letter describing the survey and were interviewed by phone within the following two weeks. This study was approved by the Committee on Ethics of the Helsinki and Uusimaa Hospital District.

### Methods

The hospital`s patient data system provided medical records and ERCP procedure information.

ERCP procedures were performed with a guidewire and a regular sphincterotome (in most of the cases Ultratome®, Boston Scientific, Miami, FL). The TPBS was performed towards the axis of the CBD, in the 11 or 12-o’clock direction. The length of the cut was the same as for a regular biliary sphincterotomy when removing small (< 1 cm) biliary stones. If needed, additional NK cut was performed obliquely towards 10 o’clock, starting from the upper end of the previous pancreatic sphincterotomy. The intention was to cut the CBD and expose the lumen.

Collected were the following patient demographic data: age, sex, BMI (body mass index), ASA (American Society of Anesthesiology Physical Status Classification) and laboratory results (bilirubin, plasma amylase before and 4 h after ERCP). Additionally collected were ERCP procedure data: cannulation method, duration of the ERCP and performed procedures. ERCP procedures were graded by the complexity of procedures criteria [20]. If a patient stayed overnight at hospital, plasma amylase was assayed 24 h after ERCP. Defining PEP occurred when plasma amylase levels were at least three times the ULN (upper limit of normal) 24 h after ERCP and when new or worsening abdominal pain with prolonged hospitalization lasted for at least two days [9, 12]. Other complications (bleeding, infection, cholangitis and perforation) were classified with the same consensus criteria and severity [9]. Late-onset AP was defined if a patient suffered from AP between 3 and 30 days after the ERCP procedure.

A phone survey collected follow-up data: recurrence of biliary symptoms (typical upper abdominal pain or diagnosed new CBD stones), episodes of AP, abdominal pain during the previous year, use of medication (painkillers, proton pump inhibitors (PPIs), pancreatin), use of alcohol and tobacco, symptoms related to pancreatic insufficiency such as steatorrhea, development of CP, hospital admissions and re-ERCPs or abdominal surgical procedures. Additionally collected were the number of re-ERCPs and both indication of and procedures during ERCP. Biliary stones were defined as remnant if new ERCP was required within the same hospitalization period and less than one week after a previous ERCP; otherwise, the stones were defined as recurrent stones.

### Statistical methods

Data analysis involved IBM SPSS Statistics for Macintosh, Version 25.0. Fisher`s exact test was used for categorical variables and Wilcoxon-Mann–Whitney U test for continuous variables when comparing both the two groups and interrelations to different variables and PEP. Probability below 0.05 was considered statistically significant. Risk factors for PEP were evaluated with binary logistic regression as an odds ratio (OR) and 95% confidential interval (CI).

## Results

All the 283 ERCP procedures were therapeutic. Three patients in TPBS (one of whom with NK) and two patients in CG (one with NK) had undergone previous failed ERCP attempts: native papilla was present in 142 and 139 patients, respectively. ERCP ended with a final failure, that is no access to the biliary tract was achieved, in four cases (2.8%) in TPBS, whereas all cases in CG were successful, as defined in the patient selection, (*p* = 0.122).

Table 1 presents patient- and ERCP-related characteristics. The majority of the procedures were performed due to CBD stones. Other indications were biliary injury after cholecystectomy, biliary pancreatitis, stricture after liver transplantation and suspicion of SOD. Rare indications in TPBS included three patients with suspicion of papillary tumor or stricture, one patient with non-specific biliary dilatation, one with Mirizzi syndrome, one with choledochal cyst and one with non-specific icterus. Other rarer indications in CG included three patients with non-specified biliary dilatation, one patient with suspicion of gallbladder fistula, one with recurrent AP and one with suspicion of a liver cyst.

**Table 1:** Patient and ERCP procedure characteristics

Characteristics	TPBS n = 143	CG n = 140	p-value
Age at ERCP (years)^a^	59 (18–93)	62 (21–89)	0.418
Female sex	106 (74.1)	99 (70.7)	0.595
BMI (kg/m^2^)^a^	26.0 (16.9–70.3)	27.9 (16.0–47.9)	0.222
ASA grade			0.272
1	24 (16.8)	22 (15.7)	
2	60 (42.0)	51 (36.4)	
3	49 (34.3)	62 (44.3)	
4	10 (7.0)	5 (3.6)	
Final failure	4 (2.8)	0 (0.0)	0.122
Indication for ERCP			
Biliary stone	112 (78.3)	104 (74.3)	0.485
Biliary stricture	15 (10.5)	11 (7.9)	0.538
Bile duct injury after cholecystectomy	15 (10.5)	16 (11.4)	0.851
Biliary pancreatitis	18 (12.6)	24 (17.1)	0.318
Problems (stricture) after liver transplantation	4 (2.8)	7 (5.0)	0.376
Other	7 (4.9)	7 (5.0)	1.000
Suspected SOD	6 (4.2)	1 (0.7)	0.120
ERCP as an emergency treatment	36 (25.4)	46 (32.9)	0.236
Periampullary diverticulum	23 (16.1)	15 (10.7)	0.223
Therapy			
Biliary sphincterotomy	141 (98.6)	138 (98.6)	1.000
Biliary stone removal	108 (75.5)	104 (74.3)	0.891
TBPS	143 (100.0)	0 (0.0)	0.000
Biliary stent placement	10 (7.0)	13 (9.3)	0.520
Biliary dilatation	6 (4.2)	6 (4.3)	1.000
Pancreatic stent placement (prophylactic)	6 (4.2)	0 (0.0)	0.030
Single operator cholangioscopy	0 (0.0)	1 (0.7)	0.495
ERCP grade of difficulty			0.133
1	4 (2.8)	4 (2.9)	
2	125 (87.4)	131 (93.6)	
3	14 (9.8)	5 (3.6)	
4	0 (0.0)	0 (0.0)	
Pancreatic duct cannulation/wire in pancreatic duct			0.000
0	0 (0.0)	113 (80.7)	
1	29 (20.3)	17 (12.1)	
2	30 (21.0)	5 (3.6)	
≥ 3	84 (58.7)	5 (3.6)	
Normal plasma bilirubin pre-ERCP	71/140 (50.7)	71/133 (53.4)	0.717
Hospitalization (nights)^a^	1 (0–65)	1 (0–16)	0.358
Operator an expert endoscopist	139 (97.2)	130 (92.9)	
Operator other/trainee	4 (2.8)	10 (7.1)	

Data are presented as numbers (n) and percentages (%) of patients or as ^a^median (range)

*TPBS* transpancreatic biliary sphincterotomy, *CG* control group, *ERCP* endoscopic retrograde cholangiopancreatography, *BMI* body mass index, *ASA* Physical status classification, American society of Anesthesiologists, *SOD* Sphincter of Oddi dysfunction

**Fig. 1:**
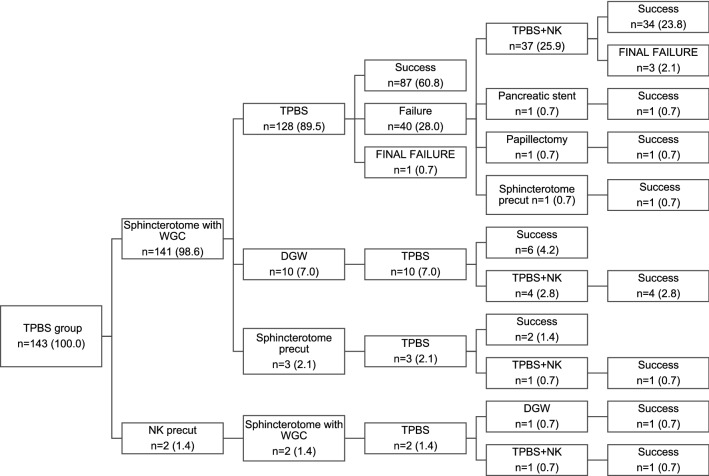
Data are presented as number (n) of patients and percentages (%). TPBS transpancreatic biliary sphincterotomy, WGC wire-guided cannulation, NK needle knife, DGW double guidewire

**Fig. 2:**
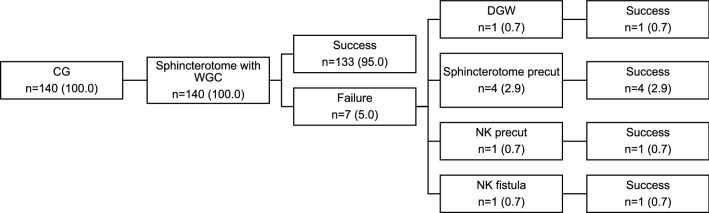
Data are presented as number (n) of patients and percentages (%). CG control group, WGC wire-guided cannulation, DGW double guidewire, NK needle knife

The primary cannulation method in the TPBS group was an NK precut in two cases (1.4%) due to a stone in the papilla. After precutting, access was possible only to PD, thus requiring further TPBS. Cannulation methods in TPBS are presented in Fig. 1 and in CG in Fig. 2.

The median duration of ERCP procedure was 27 min (7–87 min) in TPBS and 15 min (7–65 min) in CG (*p* < 0.001).

Biliary sphincterotomy (BS) was performed 141 times (98.6%) in TPBS and 138 times (98.6%) in CG (*p* = 1.000). One case in the TPBS group had no access to CBD, only to PD, and resulted in final failure after bleeding. One case was unsuccessful in TPBS + NK, although successful performance of ERCP and BS occurred three days later. In CG, one patient had suspected biliary leakage; however, due to both normal findings and normal outflow of contrast medium, BS was not performed. BS was not performed in another case involving a young patient (33 years).

The majority of procedures, that is 125 (87.4%) of TPBS and 131 (93.6%) of CG, were classified as difficulty grade 2 (20).

Table 2 lists the complications related to ERCP procedures. PEP rates were 4.9% in TPBS and 0.7% in CG (*p* = 0.067, OR for TPBS 7.15, 95% CI for OR 0.869–58.93). Complications related to anesthesia or patients’ comorbidities were pneumonia, acute transient brain ischemic attack and worsening of chronic liver dysfunction. One patient in both groups suffered from late-onset AP, both of whom were classified as mild.

**Table 2:** Complications of ERCP procedures

Complications	TPBS *n* = 143	CG *n* = 140	*p*-value
PEP	7 (4.9)	1 (0.7)	0.067
Mild	2 (1.4)	1 (0.7)	
Moderate	3 (2.1)	0 (0.0)	
Severe	2 (1.4)	0 (0.0)	
Cholangitis	1 (0.7)	1 (0.7)	1.000
Perforation	3 (2.1)	1 (0.7)	0.622
(a) Biliary tract	2 (1.4)	1 (0.7)	
(b) Bowel	1 (0.7)	0 (0.0)	
Bleeding	2 (1.4)	2 (1.4)	1.000
Other	3 (2.1)	0 (0.0)	0.247

Data are presented as numbers (*n*) and percentages (%) of patients

*ERCP* endoscopic retrograde cholangiopancreatography, *TPBS* transpancreatic biliary sphincterotomy, *CG* control group, *PEP* post-ERCP pancreatitis (classification according to Cotton et al. [9])

Table 3 presents the PEP rate related to known PEP-risk factors and incidence of PEP. According to these factors, no significant difference existed between the groups. One male and six female patients in the TPBS group suffered from PEP. None of the previously known risk factors for PEP [12, 18, 19] were significant in this study (data not shown).

**Table 3:** Incidence of post-ERCP pancreatitis according to risk factors

Risk factors	TPBS *n* = 7	CG *n* = 1	*p*-value
Female sex	6/106 (5.7)	1/99 (1.0)	0.452
Age < 40 years	2/24 (8.3)	0/22 (0.0)	0.620
Female < 40 years	2/18 (11.1)	0/13 (0.0)	0.449
History of AP	0/7 (0.0)	0/1 (0.0)	1.000
Normal serum bilirubin	4/67 (6.0)	0/62 (0.0)	1.000
ERCP duration > 40 min	3/32 (9.4)	0/7 (0.0)	0.097
Pancreatic duct opacification	0/8 (0.0)	0/0 (0.0)	1.000
NK precut	0/7 (0.0)	0/1 (0.0)	1.000
Sphincterotome precut	0/7 (0.0)	0/1 (0.0)	1.000
Suspected SOD	1/6 (16.7)	0/1 (0.0)	0.184
Biliary stone extraction	6/108 (5.6)	1/104 (1.0)	0.684
Biliary sphincterotomy	7/141 (5.0)	1/138 (0.7)	1.000

Data are presented as numbers (*n*) of post-ERCP pancreatitis patients with risk factor in number (*N*) of patients with particular risk factor and percentages (%)

*ERCP* endoscopic retrograde cholangiopancreatography, *TPBS* transpancreatic biliary sphincterotomy, *CG* control group, *AP* acute pancreatitis, *NK* needle knife, *SOD* sphincter of Oddi dyskinesia

CP was diagnosed only in one patient (0.7%) in CG and none in TPBS, (*p* = 0.495). Table 4 presents patient characteristics and phone survey data. The median follow-up period was six years in TPBS and seven years in CG.

**Table 4:** Patient characteristics at phone survey and follow-up data

Characteristics	TPBS *n* = 143	CG *n* = 140	*p*-value
Age at phone survey (years)^a^	66 (25–101)	69 (28–96)	0.253
Follow-up period (years)^a^	6 (4–10)	7 (4–10)	0.016
Cigarette smoking	14 (9.8)	11 (7.9)	0.677
Cigarettes^a^/day	15 (6–30)	10 (1–20)	0.061
Alcohol consumption	62 (43.7)	64 (46.0)	0.720
Comorbidities	117 (81.8)	112 (80.0)	0.763
Cardiovascular disease	77 (53.8)	78 (55.7)	0.811
Diabetes	21 (14.7)	25 (17.9)	0.521
Chronic lung disease	24 (16.8)	15 (10.7)	0.168
Musculoskeletal disease	44 (30.8)	44 (31.4)	1.000
Cerebrovascular disease	13 (9.1)	12 (8.6)	1.000
Gastrointestinal diseases	34 (23.8)	25 (17.9)	0.244
Endocrine diseases	10 (7.0)	27 (19.3)	0.003
Urinary tract/kidney diseases	11 (7.7)	7 (5.0)	0.466
Neurological/psychiatric disease	11 (7.7)	8 (5.7)	0.636
Other chronic disease	7 (4.9)	7 (5.0)	1.000
Cholecystectomy	118 (82.5)	117 (83.6)	
AP episodes during the follow-up period			0.238
0	140 (97.9)	134 (95.7)	
1	2 (1.4)	5 (3.6)	
2	1 (0.7)	0 (0.0)	
≥ 3	0 (0.0)	1 (0.7)	
Development of CP	0 (0.0)	1 (0.7)	0.495

Data are presented as numbers (*n*) and percentages (%) of patients or as ^a^median (range)

*TPBS* transpancreatic biliary sphincterotomy, *CG* control group, *AP* acute pancreatitis, *CP* chronic pancreatitis

Three patients (2.1%) in TPBS and six patients (4.3%) in CG suffered from AP during the follow-up period (*p* = 0.238). Abdominal pain was a common symptom in both groups: 58 patients (40.6%) in TPBS and 50 patients (35.7%) in CG mentioned upper abdominal pain in the phone survey. Of these patients, ten (6.9%) in TPBS and twelve (8.6%) in CG suffered daily from abdominal pain, while six patients (4.2%) in TPBS and twelve patients (8.6%) in CG suffered weekly. Abdominal pain was categorized as heartburn by 32.1% vs. 30.0%, as upper abdominal pain by 32.1% vs. 22.0%, as biliary-attack-type pain by 28.3% vs. 22.0% and as other unspecific pain by 7.5% vs. 26.0% in TPBS and CG, respectively.

In TPBS, 30 patients (21.1%) and in CG 22 patients (15.7%) had taken some medication for abdominal pain or symptoms within the previous year. The most common medications used were PPIs, metamizole-pitofenone, paracetamol and NSAIDs (non-steroidal anti-inflammatory drugs).

Our evaluation pertained to abdominal pain-related contacts to hospitals after index ERCP. Re-ERCP data are shown in Table 5. In all cases, biliary stones were the most common indication of re-ERCPs; however, two cases in TPBS and one case in CG concerned residual stones. Recurrent stones with gallbladder in situ occurred in three cases in both groups. Also application, exchange or removal of biliary or pancreatic stents were a common re-ERCP indication. The group “other” included six ERCPs in CG; one patient underwent four different re-ERCPs due to post-pancreatoduodenectomy stricture. Two other re-ERCPs were due to icterus (suspicion of papillary tumor) in one case and recurrent APs (and later diagnosed as CP) in one case.

**Table 5:** Re-ERCPs during the follow-up period

	TPBS	CG	p-value
Number of patients *n* (%)	18 (12.6)	7 (5.0)	0.035
Number of re-ERCPs	29	13	0.018
None	125 (87.4)	133 (95.0)	
Once	11 (7.7)	5 (3.6)	
Twice	3 (2.1)	0 (0.0)	
Three times	4 (2.8)	1 (0.7)	
Five times	0 (0.0)	1 (0.7)	
Indications for re-ERCPs	*n* = 29	*n* = 13	0.002
Biliary stones	10/29 (34.5)	4/13 (30.8)	
Biliary stricture, stent exchange /apply /removal	8/29 (27.6)	1/13 (7.7)	
Previous ERCP complication (bleeding or perforation)	4/29 (13.8)	0 (0.0)	
Pancreatic stent exchange/apply/removal	4/29 (13.8)	2/13 (15.4)	
Biliary injury after cholecystectomy	3/29 (10.3)	0 (0.0)	
Other	0/29 (0.0)	6/13 (46.2)	

Data are presented as numbers (*n*) and percentages (%) of patients

*TPBS* transpancreatic biliary sphincterotomy, *CG* control group, *ERCP* endoscopic retrograde cholangiopancreatography

Performance of re-ERCPs with an acute indication (*p* = 0.505) numbered 16 in TPBS, and 5 in CG. Other re-ERCPs were scheduled control procedures.

Patients who underwent gastroscopy during the follow-up period numbered 41 patients (28.7%) in TPBS and 46 patients (32.9%) in CG (*p* = 0.445), of whom normal findings occurred in 31 (75.6%) and 30 (65.2%), respectively.

## Discussion

We aimed to compare short- and long-term complications between TPBS and CG with native papilla and similar baseline characteristics. The follow-up period was median of 6 years in TPBS and 7 years in CG. All 283 patients were interviewed by phone, with a high participation rate: 84.1% in TPBS and 82.6% in CG. Our study provides a long follow-up period and is thoroughly performed with a matched control group and phone call survey. TPBS had acceptable cannulation success rate of 97.2%.

The criteria of difficult CBD cannulation by the Scandinavian Association for Digestive Endoscopy (SADE) research group and later by ESGE was published in 2014 and 2016 [1, 2]. The patients in our study underwent ERCPs between 2007 and 2013, when the criteria for difficult cannulation were unclear. However, patients in this study needing TPBS for successful CBD cannulation fulfilled the requirements of difficult cannulation in a retrospective evaluation. Fulfilling the requirements mostly related to PD passages, as seen in Table 1. In addition to guidewire passages to PD, the median length of the procedure was significantly longer in TPBS group, (27 min), twice as long as in CG group (15 min), indicating the difficult cannulation situation. There were no differences in performed procedures after cannulation, thus the longer procedure time was related to more time spent to cannulate. It is impossible, however, to determine the exact cannulation times and attempts retrospectively.

Only few studies have evaluated the long-term complication risks of TPBS [22, 23] or pancreatic sphincterotomy (PS) when PS was performed to gain the access to PD [24, 25]. As stated in a recent meta-analysis, long-term follow-up studies in TPBS are lacking [13]. In these studies, the median follow-up period ranged from four to five months in TPBS and 41 months in PS. Results mainly concentrate on AP or other acute complications [5, 22–26]. However, these studies have several limitations. Miao et al. followed 36 patients with TPBS for four months [22]. During that period, patients had no recurrent biliary stones or symptoms related to SOD. Kahaleh et al. evaluated 116 TPBS patients in a prospective study with a follow-up median of five months but results concentrated on short-term complications [23]. In a retrospective study, Park el al. included only SOD patients with both BS and PS with median follow-up of 41 months, the long-term outcome being a need for reintervention [25]. Kozarek et al. included only patients with CP and PS, 14% of whom required repeated endoscopic or surgical sphincter section [24]. These studies are not fully comparable to ours due to the shorter follow-up period and different patient group. Patients undergoing ERCP with symptoms related to SOD or CP are a different group than our patients who underwent ERCP for benign indications and targeting to CBD. PEP rate in this meta-analysis was 8.1% in prospective studies [13], and it is comparable to our PEP rate of 4.9%.

We have performed TPBS in difficult cannulation situation for several years and collected the data to provide a large patient series to confront the long-term complication issue. We wanted to explore if patients with TPBS have more CP or symptoms related to papillary stenosis, for example AP episodes. Therefore, we needed the follow-up period to last for several years. However, we found no relations between TPBS and CP or papillary stenosis. Concerns with TPBS included increased risk of complications, additionally long-term consequences of PS in cases when the target is not PD, and a risk of papillary stenosis that may lead to recurrent pancreatitis [27–29].

Late complications like papillary stenosis and ductal strictures are only encountered at least 3 months after the procedure indicating a need for long term follow-up [30].

In difficult cannulation, if advanced cannulation methods, such as TPBS, are needed, the risk of PEP arises [1, 2]. By the ESGE [1], TPBS should be considered as a rescue method in difficult cannulation but performed by experienced endoscopists due to its higher complication rates. Training of the TPBS technique should be focused only for trainees already being able to practice standard ERCP independently. Testoni et al., in their algorithm of biliary cannulation, placed TPBS as a third or a fourth cannulation method after DGW and/or NK precut techniques [31]. Similarly in ESGE guidelines, TPBS is recommended in difficult cannulation cases after failed attempts with standard or DGW cannulation. Sugiyama et al. performed a randomized study and suggested that TPBS should be considered as a rescue method and performed earlier in difficult cannulation [32]. As shown with our data, TPBS is safe also on a long-term scale. Therefore, we also suggest earlier performance of TPBS in difficult cannulation.

In HUS, prophylactic pancreatic stents were seldom used during the study period. The ESGE has suggested that placement of prophylactic stents might reduce the PEP rate [1, 12]. Six patients (4.2%) in TPBS had prophylactic pancreatic stent in our study (Table 1). Still, in this study, a PEP rate of 4.9% in the whole TPBS group is acceptable. At the moment, there are no clear data that after proper PS prophylactic pancreatic stent is beneficial (12) but randomized trials are needed.

Our follow-up study found no patients with stricture or CP in TPBS group. Notable, however, are limitations regarding patient selection and retrospective manner. Patients in both groups suffered from upper abdominal pain. However, no differences existed in the frequency or severity of the pain. Most of the patients had symptoms related to recurrent biliary stones or heartburn. Painkillers or PPIs were equally used in both groups. The most common reason for evaluating the follow-up contacts to healthcare were upper abdominal pain and symptoms related to biliary stones. Due to these symptoms, the most common procedures were cholecystectomy and gastroscopy.

According to a previous study in HUS, the TPBS method has been successful and is therefore commonly used as a rescue method in our unit [7]. We suggest further prospective studies to evaluate the long-term risks of TPBS.

## Limitations of the study

Possible bias in this study relates to the patient selection process. We wanted to interview all the patients; therefore, the inclusion criteria considered only benign indications for ERCP. PEP rates are slightly lower in the present study than in previous studies in the HUS endoscopy unit in ERCP patients with TPBS [7, 33]. Nevertheless, malignancy is not considered a risk factor for PEP. The ESGE guidelines for indomethacin or diclofenac as a PEP prophylaxis [12] was adopted in our unit in November 2013. In this study, only four patients underwent ERCP after November 2013 and only one patient in TPBS group received prophylactic rectal diclofenac. The ESGE definition for difficult cannulation was published in year 2016 [1]. Our study included patients between years 2007 and 2013, and we started to collect the data (cannulation duration and attempts) in March 2011. However, the missing data should not affect at least the long-term complications of TPBS.

## Conclusions

TPBS is a useful rescue method in difficult cannulation, having an acceptable complication rate. No significant difference occurred between groups when evaluating the short- or long-term complications. Additionally, no significant differences occurred during the follow-up period concerning upper abdominal pain, usage of painkillers, episodes of AP or development of CP.
